# Quantifying the Extent of North American Mammal Extinction Relative to the Pre-Anthropogenic Baseline

**DOI:** 10.1371/journal.pone.0008331

**Published:** 2009-12-16

**Authors:** Marc A. Carrasco, Anthony D. Barnosky, Russell W. Graham

**Affiliations:** 1 Department of Integrative Biology, University of California, Berkeley, California, United States of America; 2 Museum of Paleontology, University of California, Berkeley, California, United States of America; 3 Museum of Vertebrate Zoology, University of California, Berkeley, California, United States of America; 4 Department of Geosciences, The Pennsylvania State University, University Park, Pennsylvania, United States of America; 5 Earth and Mineral Sciences Museum, The Pennsylvania State University, University Park, Pennsylvania, United States of America; Paleontological Institute, Russian Federation

## Abstract

Earth has experienced five major extinction events in the past 450 million years. Many scientists suggest we are now witnessing a sixth, driven by human impacts. However, it has been difficult to quantify the real extent of the current extinction episode, either for a given taxonomic group at the continental scale or for the worldwide biota, largely because comparisons of pre-anthropogenic and anthropogenic biodiversity baselines have been unavailable. Here, we compute those baselines for mammals of temperate North America, using a sampling-standardized rich fossil record to reconstruct species-area relationships for a series of time slices ranging from 30 million to 500 years ago. We show that shortly after humans first arrived in North America, mammalian diversity dropped to become at least 15%–42% too low compared to the “normal” diversity baseline that had existed for millions of years. While the Holocene reduction in North American mammal diversity has long been recognized qualitatively, our results provide a quantitative measure that clarifies how significant the diversity reduction actually was. If mass extinctions are defined as loss of at least 75% of species on a global scale, our data suggest that North American mammals had already progressed one-fifth to more than halfway (depending on biogeographic province) towards that benchmark, even before industrialized society began to affect them. Data currently are not available to make similar quantitative estimates for other continents, but qualitative declines in Holocene mammal diversity are also widely recognized in South America, Eurasia, and Australia. Extending our methodology to mammals in these areas, as well as to other taxa where possible, would provide a reasonable way to assess the magnitude of global extinction, the biodiversity impact of extinctions of currently threatened species, and the efficacy of conservation efforts into the future.

## Introduction

Species diversity in any region varies within some limits through time. Therefore, in order to measure the extent to which humans are causing a biodiversity crisis [1], [2], perhaps even a sixth mass extinction [3]–[5], it is necessary to know how biodiversity naturally fluctuates in the absence of humans and, if the diversity today falls below this pre-anthropogenic baseline, by how much. Those numbers have been difficult to estimate because of complexities in assembling the requisite data [6]–[8]. Here we provide that estimation, by utilizing 28,019 occurrences of fossil mammal species from across temperate North America to compute species-area relationships (SARs) for many pre-anthropogenic time slices and post-anthropogenic time.

The species-area relationship is one of ecology's few widely-recognized “laws” [9]–[13], which simply states that as sampling area increases, the number of species sampled increases at a regular rate. SARs are widely used in assessing and comparing species diversity [10], [12]–[20] and are generally expressed as S = cA^z^, where S = number of species, A = area sampled, and z and c are empirically derived constants that express the slope of the power function. While alternatives to the power law have been proposed [21]–[25], it remains the most frequently used in assessing species-area relationships. SARs have proven useful in comparing diversity among different regions and groups, in estimating potential extinctions given changes in area suitable for particular groups of species (though those estimations are not without controversy [4], [11], [20]), in determining baseline targets for conservation, and in island biogeography theory [10], [12]–[19], [26], [27].

**Figure 1:**
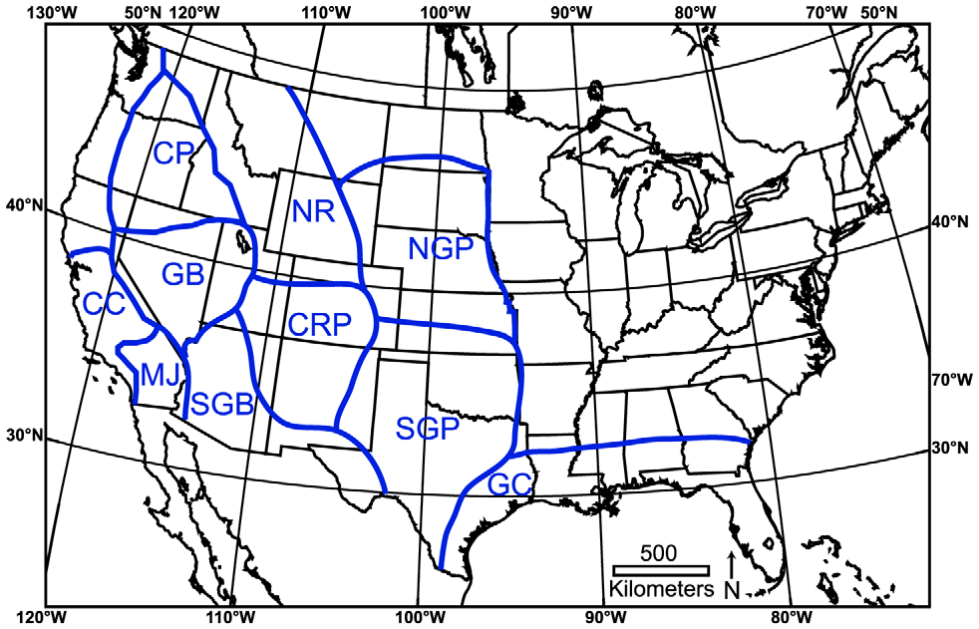
Boundaries of the ten biogeographic provinces used. Blue lines and abbreviations demarcate and label the following biogeographic provinces: CC, Central California; MJ, Mojave; CP, Columbia Plateau; GB, Great Basin; SGB, Southern Great Basin; NR, Northern Rockies; CRP, Colorado Plateau; NGP, Northern Great Plains; SGP, Southern Great Plains; and GC, Gulf Coast.

We assessed SARs for terrestrial, non-volant mammals at 19 different intervals of time (Table 1) in 10 different biogeographic provinces (Figure 1). Pre-anthropogenic time intervals were those falling between 30 million to approximately 11,500 years ago. The anthropogenic time slice was the Holocene, 11,500 to 500 calendar years ago, beginning with the first evidence for widespread humans in our study area and steepening of the human population growth curve worldwide [28]. Data were obtained from the MIOMAP database of fossil mammals for time intervals older than 5 million years and the FAUNMAP I & II databases for time intervals between approximately 5 million and 500 years ago [29], [30].

**Table 1:** Temporal Bins into Which Species Occurrences Were Sorted*.

Time Interval	Age Boundaries	Interval Duration
Holocene	∼11,500-500	∼11,000
Rancholabrean	0.15 Ma∼11,500	∼140,000
Irvingtonian	1.8-0.15 Ma	1.65 Ma
Blancan	4.7-1.8 Ma	2.9 Ma
Late Late Hemphillian	5.9-4.7 Ma	1.2 Ma
Early Late Hemphillian	6.7-5.9 Ma	0.8 Ma
Late Early Hemphillian	7.5-6.7 Ma	0.8 Ma
Early Early Hemphillian	9-7.5 Ma	1.5 Ma
Late Clarendonian	10-9 Ma	1.0 Ma
Middle Clarendonian	12-10 Ma	2.0 Ma
Early Clarendonian	12.5-12 Ma	0.5 Ma
Late Barstovian	14.8-12.5 Ma	2.3 Ma
Early Barstovian	15.9-14.8 Ma	1.1 Ma
Late Hemingfordian	17.5-15.9 Ma	1.6 Ma
Early Hemingfordian	18.8-17.5 Ma	1.3 Ma
Late Late Arikareean	19.5-18.8 Ma	0.7 Ma
Early Late Arikareean	23.8-19.5 Ma	4.3 Ma
Late Early Arikareean	27.9-23.8 Ma	4.1 Ma
Early Early Arikareean	30-27.9 Ma	2.1 Ma

*Age bins prior to 1.8 Ma are subdivisions of the North American Land Mammal Ages (NALMAs) [34] while post-Blancan time interval boundaries are those used in FAUNMAP I [30].

We used only fossil data to avoid the sampling and analytical complexities of comparing fossil with modern samples. That limited our study to assessing anthropogenic influence only up to 500 years ago, thus yielding a conservative assessment of diversity decline in respect to pre-anthropogenic times. Even though no terrestrial non-volant mammals are known to have gone extinct in our study area in the past 500 years, there have been severe range reductions of many species and at least nine subspecies have gone extinct [31]. Worldwide, about 60 mammal species have gone extinct in the past 400 years [8], and some 25% of remaining species are considered under threat of extinction [32], observations which contribute to notions we are experiencing a sixth mass extinction [3], [5], [8].

We used standard, accepted techniques to adjust for the well-recognized sampling problems inherent in using fossil data to assess diversity (see Reference [17] and Materials and Methods). We computed species richness per time slice and per geographic area by rarefying the occurrence data using a richness value of 75 occurrences, and plotted these standardized richness counts against sampled area to determine paleospecies-area relationships [17]. We examined Type IV curves (unnested analyses), which simply plot species richness against geographic area for each area sampled, as well as Type I curves (nested analyses), in which smaller geographic areas are nested inside larger ones. The resulting curves provide the pre-anthropogenic baseline to which the Holocene data points can be compared.

Details of our methods are summarized below (see Reference [17] and Materials and Methods), but two points are worth special mention. First, many of the Holocene (anthropogenic time interval) samples include more species occurrences than the pre-anthropogenic samples (Table S1), which would be expected to bias the results by producing Holocene SARs with higher diversity values (see Materials and Methods). The fact that we find just the opposite—lower Holocene diversity with respect to the pre-Holocene time slices—suggests that our conclusions are particularly robust and conservative.

Second, the Holocene time interval is the shortest one by far (Table 1). This might be expected to cause comparatively low species richness values for that time interval, if species richness is influenced by accumulating more species in longer time intervals through evolution and immigration, i.e., faunal turnover through time. We did not find this to be the case. There is no correlation between bin duration and number of localities (Pearson r = −0.0568, p = 0.624,) and the lack of correlation holds for both the overall data set and within each geographic province (Figure S1 and Table S2). In part, the expectation that potentially high within-bin turnover rates, as has been suggested for some mammal groups [33], significantly inflate species diversity in longer time intervals is not upheld because the localities in each bin do not span the entire time represented by the bin [17]. That is, the time encompassed by all fossils in each bin is actually the sum of the time represented by the individual fossil localities, which is a relatively small fragment of the overall duration of the bin. Thus, number of fossil occurrences is the more important diversity bias, which our rarefaction methods have adjusted for. Additionally, biochronologic units such as we use are definable precisely because there is *not* high turnover through discrete intervals of time; the relative lack of turnover is what produces the series of biologically meaningful groupings. Others have argued that this characteristic of little change within each bin makes biochronologic units particularly well-suited to comparisons of diversity through time [34], [35]. For these reasons it seems unlikely that our results could be explained primarily as a bias related to unequal bin durations.

## Results and Discussion

### Nested Species-Area Relationships

**Figure 2:**
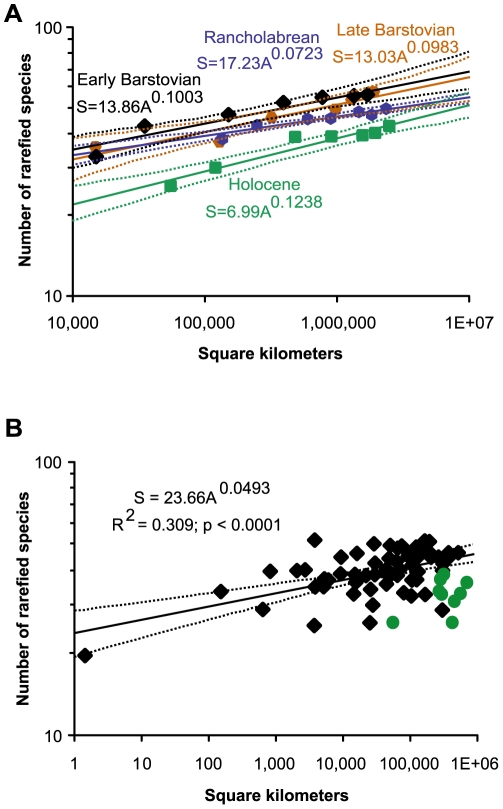
Rarefied species richness values plotted versus total geographic area through which the localities are distributed. Dashed lines represent the 95% confidence intervals for each curve. (A) Nested paleospecies area curves for the early Barstovian (black diamonds), late Barstovian (orange hexagons), Rancholabrean (blue hexagons), and Holocene (green squares) temporal intervals. (B) Unnested paleospecies area curve for all pre-Holocene (black diamonds) and Holocene time intervals (green circles). Each data point represents one biogeographic province from one temporal bin (see Table 1 for a list of temporal bins). In the equation, S = Species and A = Area.

A substantially lower SAR in the anthropogenic time interval (Holocene) is suggested by the nested analyses. Only two pre-Holocene time intervals, the early and late Barstovian (Table 1), had a sufficient number of specimens in all geographic regions to compute nested SARs (Figure 2A). The Rancholabrean (late Pleistocene) nested SAR groups with the Barstovian, but the Holocene indicates a significant drop in diversity. The fact that the early Barstovian, late Barstovian, and Rancholabrean differ in interval duration by orders of magnitude (1.1, 2.3, 0.14 million years, respectively), yet their SARs are statistically indistinguishable, makes it unlikely that interval duration could explain the difference between these three and the Holocene SAR.

In addition, it is important to note that the slopes (z) of the power law equations of the fossil Type I curves are less than those seen among modern faunas (about 0.15 [10], [13]) because of the less complete sampling of the fossil record [17]; however, the fossil samples can be evaluated relative to each other to determine how interprovincial species composition changed through the times they represent. Interestingly, the slope of the Holocene SAR is not significantly different than the slopes of the Barstovian and Rancholabrean ones, indicating that the difference in species composition between provinces changed very little, if at all, even in the face of the shift of world climate into ice ages (the Pleistocene) and the Holocene diversity decline. The slope of the Holocene SAR may also reflect extinctions that preferentially affected vulnerable species from widespread geographic ranges (e.g., large-bodied mammals). This would result in a drop in total diversity, but little change in between province diversity. A more in depth evaluation of the individual species across provinces within each time interval would be helpful in testing for this effect.

### Unnested Species-Area Relationships

Lowered Holocene diversity also is evident in comparing the Holocene SAR to the pre-Holocene SAR using an unnested approach (Figure 2B). The Holocene species richness values are consistently below those of the older temporal bins, falling short of the expected number of species at all geographic areas based on the regression line of the pre-Holocene data (black line in Figure 2B).

**Figure 3:**
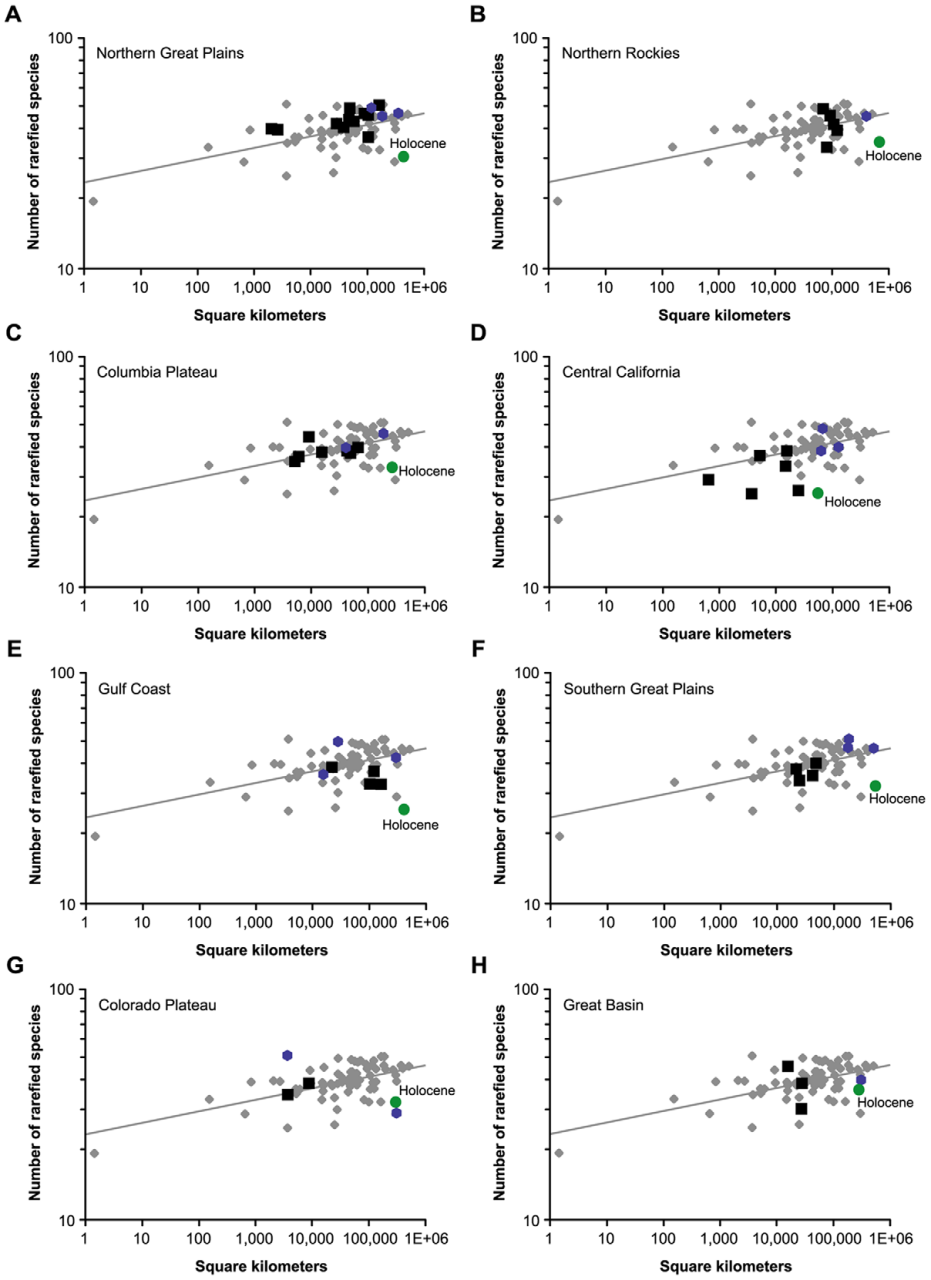
Rarefied species richness values plotted versus total geographic area through which the localities are distributed. The gray diamonds and line show paleospecies area relationships for the entire pre-Holocene data set as in Figure 2B; the black squares represent each of the respective intraprovincial data points for Oligocene and Miocene time slices (30–4.7 Ma); the blue hexagons represent each of the respective intraprovincial data points for the Blancan, Irvingtonian, and Rancholabrean time slices (4.7 Ma–11,500 years ago); and the green circle shows the Holocene time interval. (A) Northern Great Plains (NGP in Figure 1). (B) Northern Rockies (NR). (C) Columbia Plateau (CP). (D) Central California (CC). (E) Gulf Coast (GC). (F) Southern Great Plains (SGP). (G) Colorado Plateau (CRP). (H) Great Basin (GB).

Region-by-region unnested analyses show that in every biogeographic province, Holocene diversity is lower than expected relative to all within-province fossil time slices for pre-Holocene time intervals. A more detailed evaluation was made of those provinces with three or more intraprovince fossil time slices (Figure 3), inasmuch as they offer a more refined regional baseline. Of the eight provinces that satisfy this criterion, all show depressed Holocene diversity. Using the regression equation shown in Figure 2B to predict diversity values, the rarefied values for the Holocene in these eight provinces are 15–42% (avg. = 27.7%) lower than expected. Residual analyses also support the contention that the Holocene data points are generally anomalous—the four largest standard residuals (ranging from 1.56–1.72) of the baseline regression line are all from the Holocene. As in the nested continental analysis (Figure 2A), the regional data argue against differing interval durations explaining the low Holocene diversity. In the regional SARs, the Rancholabrean interval duration (∼140,000 years) is most similar to the Holocene interval duration (∼11,000 years), yet its species-richness values are indistinguishable from the time-intervals that measure in millions of years, except in one case, the Colorado Plateau.

Rancholabrean species richness for the Colorado Plateau falls noticeably below that of the Holocene data point (also unexpected if interval duration were controlling the signal) (Figure 3G). While it is possible that the low diversity value for the Rancholabrean of the Colorado Plateau is real, closer inspection of this data point reveals an anomalous pattern of occurrences per locality. The Rancholabrean of the Colorado Plateau has only 132 occurrences, and these are spread across 47 localities—less than three occurrences per locality. Of the 47 localities, most have low fossil yields with 29 having only a single occurrence, generally a large mammal such as *Mammuthus* or *Bison*. The effect of such low fossil yields per locality, and localities spread across the province, could be to artificially depress the diversity adjusted for sampling area. Alternatively, it could be that the diversity decline became apparent slightly earlier (late Pleistocene) in the Colorado Plateau than elsewhere. Only further field work to discover and report well-sampled Rancholabrean fossil localities in the Colorado Plateau can resolve these alternatives.

### Relation to End-Pleistocene Extinction

The depression of Holocene diversity indicated by our data largely results from the well-documented extinctions of Pleistocene mammalian megafauna [28], [36]–[38], and are consistent with conclusions of many others that human impacts probably figured prominently in the extinctions [36]–[38]. The pre-anthropogenic baseline includes climatic shifts that in magnitude and rate rival those seen at the end of the Pleistocene (for example, glacial-interglacial cycles throughout the Pleistocene, and the rapid cooling at the end of the Pliocene) [38], [39], yet none of the pre-anthropogenic time slices exhibit the severe diversity depression characteristic of the anthropogenic (Holocene) one.

Likewise, our results are concordant with previously reported late Pleistocene and early Holocene range shifts that decreased small-mammal species richness in some places, attributed to end-Pleistocene climatic changes [18], [40], [41]. In all seven of the provinces for which at least 75 occurrences of small-bodied taxa (the sum of Rodentia, Lagomorpha, and Insectivora species) were available, rarefied diversity decreases across the Rancholabrean/Holocene boundary (Table 2).

**Table 2:** Rarefied Species Diversity Values and Number of Occurrences of Small Mammals for the Rancholabrean and Holocene Time Intervals*.

Biogeographic Province	Rancholabrean	Holocene
Central California	28.6 (203)	25.8 (208)
Great Basin	31.1 (206)	30.5 (544)
Gulf Coast	28.0 (384)	21.0 (329)
Northern Great Plains	38.9 (255)	23.5 (447)
Northern Rockies	32.2 (207)	28.8 (737)
Southern Great Basin	35.6 (349)	30.5 (284)
Southern Great Plains	35.5 (562)	27.2 (556)

*Only those provinces for which greater than 75 occurrences were available for both time periods are listed. Rarefied species numbers are per province per time slice and were determined following the procedures outlined in the text.

These results are consistent with the idea of synergy between human impacts and end-Pleistocene climate being the underlying driver of the initial Holocene diversity reduction [38], [42], [43].

### Conclusions

Our results provide a quantitative assessment of what has long been primarily a qualitative observation: namely, the decline in mammal diversity that occurred as human presence first began to dominate the North American landscape. We demonstrate that this decline represented a 15–42% loss (depending on biogeographic province) in mammal species richness. Therefore, the diversity baseline we are at today already is well below the “normal” biodiversity baseline for North American mammals, if we define “normal” as the condition that prevailed through most of the millions of years modern mammal families have been on Earth. In that light, the current indications that extinction of mammals may be accelerating in North America, as evidenced by the historic loss of at least nine subspecies and severe historic range reductions of many species [31], is of special concern because future losses would be from a fauna that already has been depressed well below normal diversity levels.

It is not yet possible to quantify diversity loss using the same metrics for mammals of other continents or for other types of organisms. Nevertheless, it is informative to compare the diversity decline we document for North American mammals with species losses that characterize the five mass extinctions recognized in the geological record. Each of those mass extinctions resulted in the loss of at least 47±4.1% of the known genera living on Earth at their respective times (about 55% according to the recent analysis of Alroy et al. [44] of marine invertebrates), which has been extrapolated to a loss of at least 76±5% of the known species [6]; 75% species loss thus seems a reasonable benchmark for defining a mass extinction. Our data show that North American mammals have already progressed at least one fifth and perhaps more than halfway towards that benchmark.

We recognize that mammal diversity declines likely differ in magnitude from diversity reduction in other kinds of species, and other continents may ultimately exhibit different magnitudes of diversity loss than North America. However, we note that major species losses of mammals on other continents have been widely recognized in qualitative terms [37], [38], [42], [45], and globally mammals [32] as well as other well-studied groups evidence major species losses over historic time [5], [8]. Sufficient data are on the verge of becoming available in South America and Australia, but will await compilation into requisite electronic databases. In Eurasia, similar analyses are in theory possible by utilizing existing databases such as the Neogene of the Old World database [46]. Therefore, methods such as we employ here may well be useful in assessing extinction magnitudes in these other places and taxa, which is needed to conclusively determine how far we have progressed towards a sixth mass extinction on a global basis.

Finally, our results also define a “new normal” diversity baseline for mammals of temperate North America, the Holocene one from which all future mammal communities will evolve. We suggest that continued refinement of present day SARs for mammals and for as many other taxa as possible, such that there is a robust baseline against which to compare future diversity patterns, will provide a valuable assessment tool to recognize the extent to which conservation efforts are effective at stemming or reversing the diversity decline we document here.

## Materials and Methods

Species occurrence data were extracted from the MIOMAP [29], FAUNMAP I [30], and FAUNMAP II (available soon through [29] or from the authors) databases.

### Biogeographic Provinces

Today the regions in Figure 1 are considered biogeographically distinct from one another [47]–[50] and it is likely that the same held true back through the Oligocene [34], [41], [51]–[53]. This is particularly true in those provinces that have the most complete fossil record (e.g., Northern Great Plains, Southern Great Plains, Gulf Coast) as they have undergone limited topographic change over the past 30 million years [54], [55].

### Adjusting for Biases in Fossil Samples

There are well-recognized sampling problems that must be adjusted for when using fossil data to assess diversity because the number of samples and geographic area sampled per time slice is widely variable and because the time slices are unequal in duration. To adjust for the potential bias of unequal sample size, we computed species richness values per time slice and per geographic area by rarefying the data using a richness value of 75 taxon occurrences (whether a taxon was present or absent at a given locality). We plotted these standardized richness counts against geographic area to determine paleospecies-area relationships. Geographic areas were calculated by using the Berkeley Mapper mapping interface (http://berkeleymapper.berkeley.edu) to zoom in on the set of localities at appropriate scales, trace the minimum convex polygon that would enclose all the localities of interest within a particular province, and calculate the area enclosed by the polygon. The SARs were evaluated by constructing both Type I and Type IV curves (see Scheiner [12] for an explanation of these curves) following the methods outlined in reference [17].

The problem with how to deal with unequal time slices arises because relatively few fossil localities can be precisely dated, which means that at best fossils can be lumped only into broad intervals of time in deposits older than the effective range of radiocarbon dating (near 50,000 years). Methods used to temporally sort the fossils include radiometric dating techniques, such as Ar-Ar dating of the fossil-bearing rocks, or relative dating tied to radiometric dates, such as magnetostratigraphy, or biochronologies determined using the evolutionary stage of the fossils themselves. Innovative algorithms based on taxon co-occurrences have been developed to sort fossil occurrences into equal 1 million year intervals [56]–[60], but were inappropriate in our study because they can introduce false precision and reduce the number of localities per time slice so that no data exists for many time slices when dividing the record into several different biogeographic regions. Because of that data limitation, we use the divisions and subdivisions of the North American Land Mammal Ages (NALMAs) [30], [34]. As documented in Table S2 and Figure S1, the unequal bin durations of the subdivided NALMAs do not seem adequate to explain our results because there is no correlation between bin duration and species richness, the actual length of time represented by the fossil localities in the bins is much less than the overall bin duration, and because each NALMA subdivision is by definition a time interval of relatively little faunal turnover. The anthropogenic (Holocene) and pre-anthropogenic time intervals are similar subsets of the total fauna of non-volant terrestrial mammals as represented by voucher specimens in museum collections. Samples from all time slices were collected primarily over the past century by various scientists usually employing non-random collecting methodologies (e.g., near main roads or home institutions, accessible habitats, etc.). However, Holocene samples are often larger, which would be expected to result in a greater number of total species in each biogeographic province. Because correlation analyses indicate a significant correlation within each bin between the total number of species (Table S2) and the rarefied species richness (Pearson r = 0.5654, p<0.0001) as well as the total number of occurrences (Table S1; Pearson r = 0.6739, p<0.0001), the SARs from the anthropogenic bin should have higher diversity if this bias were influencing our conclusions. They do not; in fact, they are lower.

### Species Counts

Two alternative methods for calculating the number of species per geographic area are maximum (regards all specimens that are only identified to genus or higher taxon as belonging to unique species) and minimum counts (regards all specimens only identified to genus or higher taxon as belonging to a species represented by more diagnostic material). Only minimum counts were employed here as previous work has shown little difference among them [17].

### Rarefaction Methods

Rarefaction of the raw minimum species counts was accomplished with S. Holland's analytic rarefaction software (http://www.uga.edu/~strata/software/). A review of the development of rarefaction methodology can be found in Tipper [61]; the programs we used were ultimately based on the analytical solutions to rarefaction presented by Raup [62], and originally derived by Hurlbert [63] and Heck et al. [64]. The data was rarefied by occurrences instead of the number of individual specimens to remove the effect of high-graded localities and missing data [17]. We set the rarefaction occurrence value at 75 because that occurrence value provided a large number of data points while at the same time eliminating points that were based on suspect, spotty data. Rarefaction of only the small mammals of the Rancholabrean and Holocene time intervals was done in an identical manner, but only taxa of the Rodentia, Insectivora, and Lagomorpha were used in the analyses.

## Supporting Information

Figure S1 Relationship between interval length and number of species recorded in each temporal bin by biogeographic province. Note that there is no correlation between interval length and number of species, either within biogeographic provinces or overall. Biogeographic province abbreviations follow those in Figure 1.
(7.92 MB TIF)

Table S1 Number of Occurrences for Each Temporal Bin by Biogeographic Province.
(0.06 MB DOC)

Table S2 Number of Total Species for Each Temporal Bin by Biogeographic Province.
(0.05 MB DOC)
