# Supplementary figures and images for: Quantifying the Extent of North American Mammal Extinction Relative to the Pre-Anthropogenic Baseline
Source: PLoS One. 2009 Dec 16;4(12):e8331. doi: 10.1371/journal.pone.0008331 (PMC2789409; doi:10.1371/journal.pone.0008331)

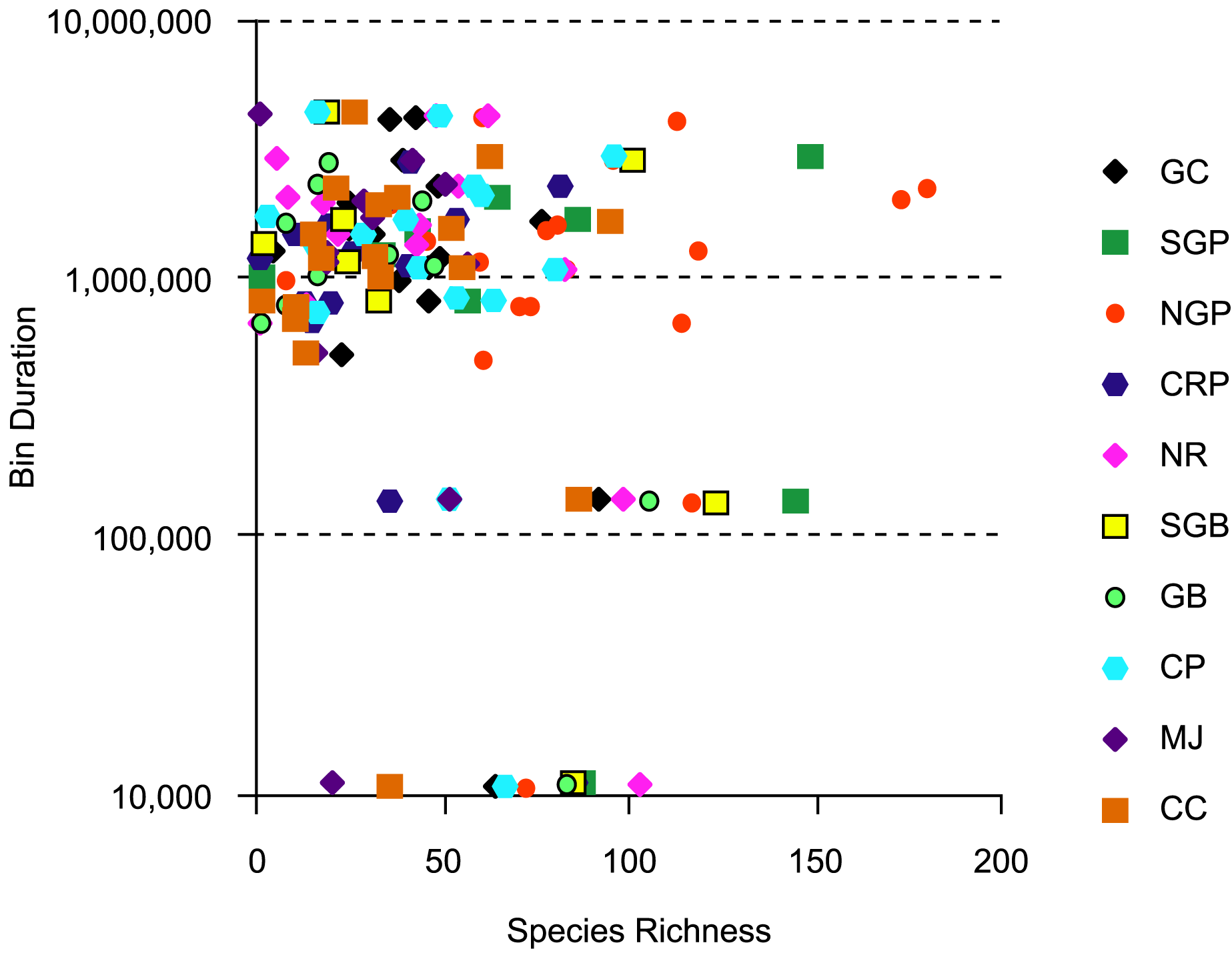

Supplement: Figure S1 — Relationship between interval length and number of species recorded in each temporal bin by biogeographic province. Note that there is no correlation between interval length and number of species, either within biogeographic provinces or overall. Biogeographic province abbreviations follow those in Figure 1. (7.92 MB TIF) [file pone.0008331.s001.tif]
