# Supplementary material for: Quantifying the Extent of North American Mammal Extinction Relative to the Pre-Anthropogenic Baseline
Source: PLoS One. 2009 Dec 16;4(12):e8331. doi: 10.1371/journal.pone.0008331 (PMC2789409; doi:10.1371/journal.pone.0008331)
Supplement: Table S1 — Number of Occurrences for Each Temporal Bin by Biogeographic Province. (0.06 MB DOC) [file pone.0008331.s002.doc]

| **Age** | **CC** | **MJ** | **CP** | **GB** | **SGB** | **NR** | **CRP** | **NGP** | **SGP** | **GC** |
| --- | --- | --- | --- | --- | --- | --- | --- | --- | --- | --- |
| HOLO | 208 | 57 | 567 | 798 | 462 | 1462 | 1582 | 1000 | 1031 | 865 |
| RANCH | 494 | 144 | 91 | 507 | 669 | 525 | 132 | 446 | 1061 | 991 |
| IRVI | 245 | - | 2 | - | 37 | - | 83 | 195 | 250 | 160 |
| BLAN | 199 | 69 | 529 | 23 | 354 | 10 | 73 | 327 | 435 | 92 |
| LLHP | 36 | 81 | - | 132 | 42 | - | 1 | 103 | 81 | 254 |
| ELHP | 1 | - | 145 | 9 | 64 | - | 21 | 176 | 140 | - |
| LEHP | 13 | - | 190 | 1 | - | 26 | 18 | 212 | 62 | 114 |
| EEHP | 17 | - | 82 | - | 18 | 25 | 25 | 92 | 119 | 75 |
| LCLA | 64 | - | 131 | 19 | - | - | - | 14 | 1 | 39 |
| MCLA | 120 | 47 | - | 97 | - | 26 | 54 | 55 | 222 | 37 |
| ECLA | 19 | 19 | - | - | - | - | - | 298 | - | 44 |
| LBAR | 48 | 228 | 129 | 23 | - | 146 | 320 | 1334 | - | 185 |
| EBAR | 225 | 218 | 349 | 85 | - | 178 | 99 | 443 | - | 125 |
| LHMF | 129 | 54 | 66 | 8 | - | 198 | 28 | 291 | - | 33 |
| EHMF | 92 | 29 | 24 | - | 2 | 77 | 53 | 729 | - | 5 |
| LLAK | 19 | - | 32 | - | - | 1 | 19 | 399 | - | - |
| ELAK | 85 | 5 | 36 | - | 57 | 74 | - | 139 | - | 73 |
| LEAK | - | - | 117 | - | - | 133 | - | 322 | - | 73 |
| EEAK | 79 | - | 189 | - | - | 9 | - | 857 | - | - |

Age abbreviations are as follows: HOLO, Holocene; RANCH, Rancholabrean; IRVI, Irvingtonian; BLAN, Blancan; LLHP, Late Late Hemphillian; ELHP, Early Late Hemphillian; LEHP, Late Early Hemphillian; EEHP, Early Early Hemphillian; LCLA, Late Clarendonian; MCLA, Middle Clarendonian; ECLA, Early Clarendonian; LBAR, Late Barstovian; EBAR, Early Barstovian; LHMF, Late Hemingfordian; EHMF, Early Hemingfordian; LLAK, Late Late Arikareean; ELAK, Early Late Arikareean; LEAK, Late Early Arikareean; EEAK, Early Early Arikareean. Biogeographic province abbreviations follow those in Figure 1.
