# Supplementary material for: Quantifying the Extent of North American Mammal Extinction Relative to the Pre-Anthropogenic Baseline
Source: PLoS One. 2009 Dec 16;4(12):e8331. doi: 10.1371/journal.pone.0008331 (PMC2789409; doi:10.1371/journal.pone.0008331)
Supplement: Table S2 — Number of Total Species for Each Temporal Bin by Biogeographic Province. (0.05 MB DOC) [file pone.0008331.s003.doc]

| **Age** | **CC** | **MJ** | **CP** | **GB** | **SGB** | **NR** | **CRP** | **NGP** | **SGP** | **GC** |
| --- | --- | --- | --- | --- | --- | --- | --- | --- | --- | --- |
| HOLO | 36 | 21 | 68 | 83 | 86 | 103 | 86 | 73 | 88 | 65 |
| RANCH | 87 | 52 | 52 | 106 | 124 | 99 | 37 | 117 | 145 | 93 |
| IRVI | 95 | - | 2 | - | 24 | - | 55 | 81 | 86 | 77 |
| BLAN | 63 | 42 | 95 | 20 | 102 | 6 | 41 | 96 | 149 | 39 |
| LLHP | 18 | 20 | - | 36 | 24 | - | 1 | 60 | 35 | 50 |
| ELHP | 1 | - | 53 | 9 | 33 | - | 21 | 74 | 57 | - |
| LEHP | 12 | - | 63 | 1 | - | 13 | 13 | 71 | 33 | 47 |
| EEHP | 17 | - | 28 | - | 15 | 22 | 11 | 46 | 44 | 32 |
| LCLA | 33 | - | 44 | 17 | - | - | - | 8 | 1 | 39 |
| MCLA | 32 | 30 | - | 45 | - | 18 | 34 | 37 | 66 | 25 |
| ECLA | 14 | 16 | - | - | - | - | - | 61 | - | 24 |
| LBAR | 21 | 53 | 59 | 17 | - | 55 | 82 | 181 | - | 49 |
| EBAR | 55 | 58 | 78 | 49 | - | 83 | 42 | 84 | - | 47 |
| LHMF | 53 | 32 | 39 | 8 | - | 44 | 20 | 78 | - | 25 |
| EHMF | 31 | 19 | 16 | - | 2 | 43 | 26 | 119 | - | 5 |
| LLAK | 11 | - | 18 | - | - | 1 | 15 | 115 | - | - |
| ELAK | 26 | 2 | 16 | - | 19 | 49 | - | 61 | - | 43 |
| LEAK | - | - | 50 | - | - | 62 | - | 113 | - | 37 |
| EEAK | 38 | - | 61 | - | - | 9 | - | 174 | - | - |

Age abbreviations follow those in Table S1. Biogeographic province abbreviations follow those in Figure 1.
